# Seasonal patterns of long sickness absence due to 411 diagnostic groups: a nationwide register-based study in Finland during 2020–2023

**DOI:** 10.1177/14034948251327545

**Published:** 2025-08-11

**Authors:** Aapo Hiilamo, Tea Lallukka

**Affiliations:** 1 Max Planck Institute for Demographic Research, Rostock, Germany; 2 Max Planck – University of Helsinki Center for Social Inequalities in Population Health, Rostock, Germany and Helsinki, Finland; 3 Department of Public Health, Faculty of Medicine, University of Helsinki, Finland

**Keywords:** Seasonality, sickness absence, clustering

## Abstract

**Aim:**

Seasonal patterns in sickness absence (SA) are little studied but crucially important to understand in order to design preventative measures and allocate resources. We aimed to identify seasonal patterns in long-term SA, that is, absences longer than 10 working days, due to different diagnostic groups.

**Method:**

Long-term SA recipients on a monthly basis from 2020 through 2023 were analyzed (2,257,011 long-term SA recipients in total). Monthly relative deviations from the expected SA recipient numbers given no seasonality were calculated for each diagnostic group defined by digits of ICD-10 codes. The seasonal deviations in 411 different diagnoses were used as input in an unsupervised learning method, the K-means clustering algorithm, to identify specific diagnoses susceptible to seasonal variation.

**Results:**

The number of long-term SA recipients was lowest in the summer, and reached three peaks in February–March, October, and December. We identified three seasonal patterns by diagnostic group. A winter and autumn peaks cluster (6% of SA recipients) consisted of 42 diagnostic groups, such as sleep disorders. A spring high cluster (81%) included mainly mental and musculoskeletal diagnoses. An autumn high cluster (13%) consisted of a mixed set of 262 diagnostic groups, including stress-, injury-, and musculoskeletal disorder-related diagnoses. These clusters differed in terms of the age and gender of the recipients.

**Conclusions:**

**There is substantial potential to reduce SA by addressing its seasonal determinants. The identified patterns could be used to design the optimal provision of preventative measures throughout the calendar year in health policies, occupational health care, and workplaces.**

## Introduction

Mortality and morbidity follow seasonal patterns. In the north, a winter peak in mortality has been observed across countries and time periods [1–3]. The seasonal patterns are often attributed to the (cold) climate triggering biological processes, such as changes in blood pressure in cold weather, discussed in detail elsewhere [4,5]. Geographical variation and the reduction, in more recent decades, in the magnitude of the seasonality indicate that social mechanisms, like energy poverty, are relevant in the causal chains [6,7]. Seasonality of morbidity varies across health conditions. Some mental disorders, such as unipolar depression, show a peak in the first month of the year, but others follow more complex patterns [8]. The causal mechanisms explaining the seasonality of morbidity are complex. For example, the role of daylight, in general, and melatonin levels, in particular, have been proposed as possible links between the seasons and depression [9], but the evidence on these mechanisms is inconsistent [10]. Numerous plausible determinants of health are shown to vary by season including lifestyle factors, such as alcohol use [11], physical activity [12], sleep patterns [13] and diet quality [14].

Identifying seasonal patterns of diseases, in general, and work disability, in particular, are important public health tasks. First, efficient public health- and occupational health care delivery need to understand the seasonal variations in disease load in order to allocate resources, such as doctors, nurses and physiotherapists, to meet the demand for such services. Second, season-specific prevention measures are needed, for example, to prevent falls that are more prevalent in the winter in the Nordic countries [15]. Third, seasonality can help us to understand the disease loads of vulnerable population groups and thereby reduce health inequalities. Fourth, employers need information on when to expect higher levels of absenteeism to counteract these shortages.

However, it is still unclear whether the seasonality in hospitalizations and mortality can be observed for sickness absence (SA). SA, that is, temporary absence from work for health reasons, concerns working-aged populations, while the findings regarding seasonality of mortality and morbidity show that older populations are more susceptible to seasonal effects. SA predicts disability retirement [16] and mortality [17]. Moreover, SA is conceptually different from mortality and morbidity. SA is a measure of work disability, which is a mismatch between one’s health and work requirements. In many occupations, seasonality affects working conditions.

We were able to identify only one previous Nordic study on seasonal patterns in long-term SA. Virtanen et al. found that SA due to depressive, anxiety, and sleep disorders peaks at the end of the year [18]. However, this study focused exclusively on common mental disorders and used data from before the COVID-19 pandemic (2006–2017), which brought about changes in working conditions [19,20]. COVID-19 increased remote working but only in some occupations, which led to potentially polarized levels of stress, risk of infection, and travel times among workers. It is yet to be confirmed whether their findings observed before the pandemic on seasonality of SA due to mental diagnoses can be replicated after the pandemic.

It is also unclear whether different diagnostic groups follow similar seasonal patterns. Much research has focused on seasonality of morbidity in specific diseases, such as infectious diseases [21] and mental disorders [8] but there is less exposure-wide assessment. While common mental disorders, studied by Virtanen et al., account for a large share of all SA, it is important to study SA resulting from a wide range of diagnoses and their seasonal patterns.

In this study, we describe seasonal patterns in long-term SA for different diagnostic groups using Finnish full population data covering long-term SA spells in 2020–2023. We address the limitation of the existing literature on seasonality of morbidity by using this neglected yet important measure.

## Methods

We used aggregated data on SA recipients covered by the Social Insurance Institution of Finland (SII). SII typically covers SA after a 10-day waiting period (employers may cover the SA days during this waiting period), and can be paid up to 300 days. A medical certificate is required to receive payments. People aged 16–67 (including students, entrepreneurs, and the unemployed) are eligible for long-term SA. Full-time pensioners and people under age 16 are not eligible. The need for long-term SA is assessed by a health care professional, and is based the job requirements of the individual’s current or most recent occupation [22].

We used sex, age group, diagnostic group, and month–year aggregated data on long-term SA recipients between 2020 and 2023. That is to say that our study design was ecological. Our data included all persons who had an SA spell that included the 15^th^ day of month between 15 January 2020 and 15 December 2023. The main diagnoses associated with long-term SA are provided in the Finnish version of International Statistical Classification of Diseases (ICD)-10 two-digit codes. The analyzed data did not include part-time long-term SA, long-term SA for entrepreneurs, special allowances for infectious disease (this was not SA due to COVID-19 infections but a compensation for not working due to quarantine requirements), or special long-term SA paid directly to employers for employees being organ donors. The data included 1473 diagnostic groups. However, we excluded diagnostic groups with fewer than 60 recipients annually any year of the study period for two reasons. First, some diagnoses in these groups were missing due to data protection laws and regulations. Second, these diagnoses have more fluctuations due to the small recipient numbers with little population-level importance. Our analyzed data included 411 different diagnostic groups and 2,257,011 long-term SA spells in total. These diagnostic groups accounted for 97% of all long-term SA spells in our data. The same person could contribute to more than one aggregated data point if this person received SA in multiple months. However, the SA diagnostic groups in single time points are mutually exclusive.

We analyzed the seasonal trends first by the main chapters of the ICD-10 and then by more detailed second digits of the ICD-10 codes. We first calculated the monthly number of long-term SA recipients, $O_{dmy}$ , for diagnostic group, *d*, in month, *m*, and year, *y*. Next, we calculated the expected number of diagnostic group-year-specific long-term SA recipients under the assumption of no time-related variation by taking the yearly average of the observed monthly number of long-term SA recipients as follows:

$$E_{dy}=\frac{1}{12}\sum_{m=1}^{12}O_{dmy}$$

To assess seasonality, we then divided the monthly observed values by the expected values:

$${\left(O/E\right)}_{\_dmy}=\frac{O_{dmy}}{E_{dy}}$$

Finally, we averaged this over the 4-year observation period

$${\left(O/E\right)}_{\_dm}=\frac{1}{4}\sum_{y=2020}^{2023}{\left(O/E\right)}_{\_dmy}$$

Our measure thus represents the deviation from the expected monthly value averaged over 2020–2023.

For the main chapters we reported observed trends. We then identified diagnostic group-specific seasonal patterns using the K-means clustering algorithm. This is an unsupervised machine learning method to group observations by minimizing within-cluster variability [23]. Our input to the cluster analysis was monthly deviations (12 variables, one for each month), which were scaled, and the rows were diagnostic group-specific long-term SA recipients (411 diagnostic groups). We selected the optimal number of clusters based on the silhouette method [24] (see Supplementary Figure 1) and a meaningful interpretation of the groups. We then explored the age and sex of the SA recipients in each cluster. (Replication materials are provided online:https://osf.io/3rgc5/?view_only=db408a81f85041b9ba09ae8b40ac1ac4 (anonymized).)

## Results

**Figure 1.**
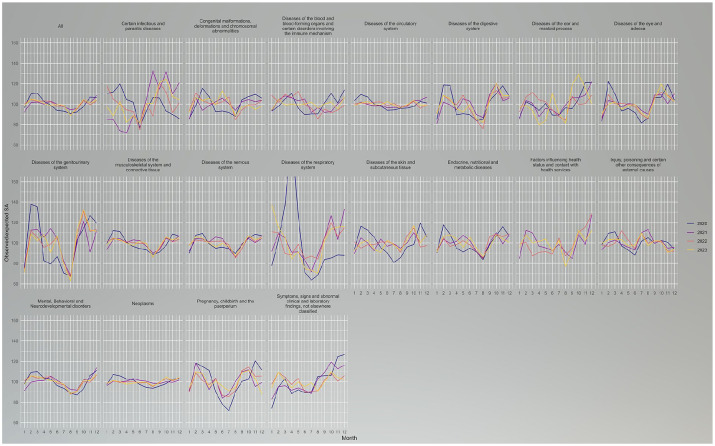
Observed/expected long-term sickness absence (SA) recipients by year, month, and ICD-10 main chapters in Finland in 2020–2023. Number of recipients measured at cross-section every 15^th^ day of the given month. *Note*: Y-axis is truncated. *Source*: Social Insurance Institution of Finland register data.

Long-term SA followed a similar three-peak seasonal pattern in all years and age groups: increasing at the start of the year, declining from May to August, increasing again in September, declining slightly in November (except in 2020), and then peaking in December, at 5% higher than the yearly average (Figure 1). Moreover, in all years there was a steep drop from December to January, which was particularly steep in 2020–2021. The ICD-10 chapter-specific trends suggest that this drop was due to steep decreases in both infections and mental disorder diagnoses. There were substantial differences in the magnitude of seasonality by ICD-10 chapter. Women had more long-term SA spells in the spring and men in August (Supplementary Figure 3) and younger recipients had more during summer than older recipients.

**Figure 2.**
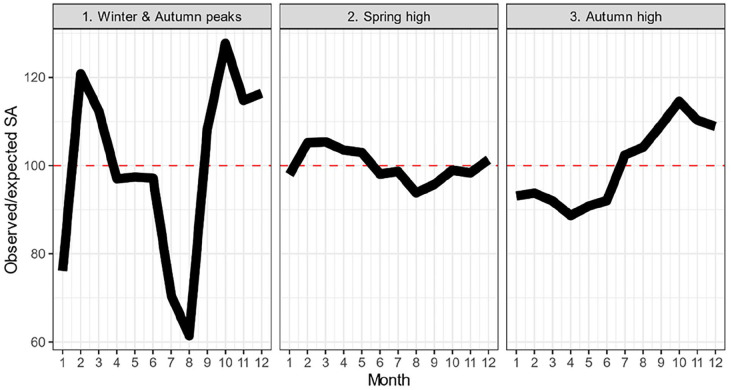
Seasonal patterns of all long-term sickness absence (SA) recipients (*N* = 2,257,011) by the identified seasonal clusters in Finland in 2020–2023. Y-axis shows the observed/expected ratio. *Note*: Y-axis is truncated. The line shows the cluster-specific average.

**Figure 3.**
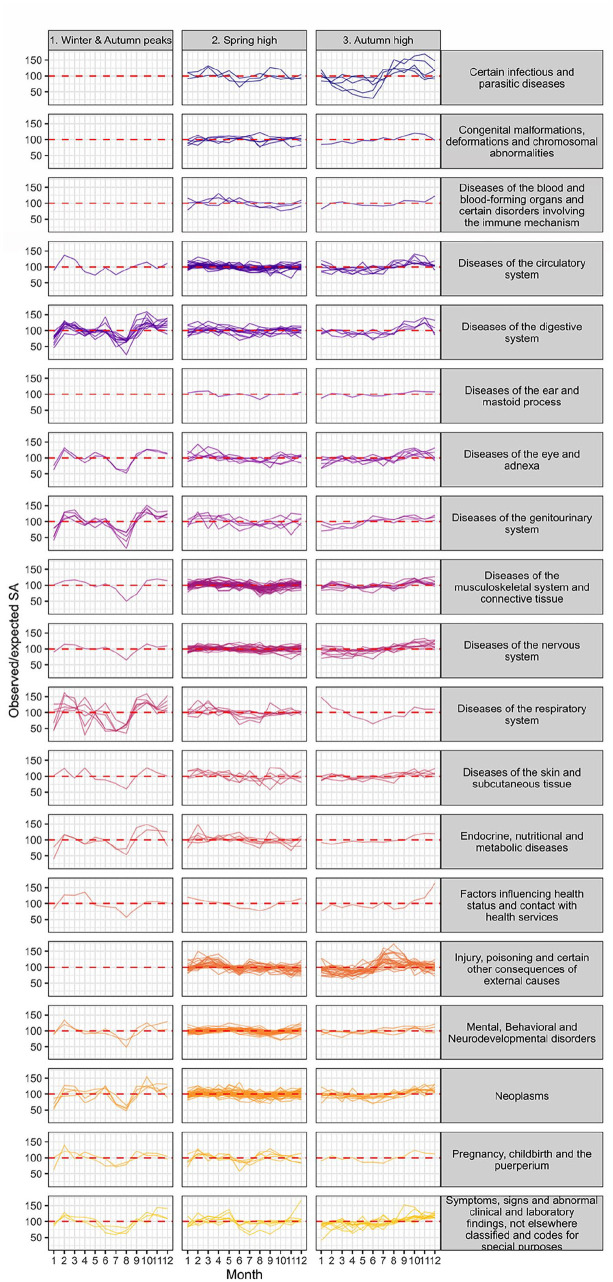
Observed/expected long-term sickness absence (SA) recipients by assigned clusters (horizontal subplots), month, ICD-10 main chapters (vertical subplots), and specific diagnostic groups (different lines) in Finland in 2020–2023. Number of recipients measured at cross-section every 15^th^ day of the given month. *Note*: Y-axis is truncated. *Source*: The Social Insurance Institution of Finland register data.

We identified three clusters of long-term SA seasonal variation in 411 different specific diagnostic groups (Figure 2) and by diagnostic group-specific trends (Figure 3). The winter and autumn peaks’ high cluster consisted of 42 diagnostic groups. There were substantially more long-term SA recipients in February–March and in September–December. This cluster included 132,326 long-term SA recipients in 2020–2023 (6% of the analyzed long-term SA recipients; Table I), some 66% of whom were women. The most significant individual diagnostic group in terms of the number of recipients was mononeuropathies of upper limb (G56) and sleep disorders (F51).

**Table I.** Total number of diagnoses, long-term sickness absence (SA) recipients by the identified clusters and most common diagnostic groups (ICD-10) in Finland in 2020–2023. K-means clustering analysis used to assign clusters. The Social Insurance Institution of Finland register data.

	All	Winter and autumn peaks	Spring high	Autumn high
Number of diagnostic groups	411	42	262	107
Number of long-term SA recipients	2,257,011	132,326	1,836,238	288,447
Distribution of long-term SA days by age	Col %	Col %	Col %	Col %
16 to 29	16	11	17	13
30 to 49	37	43	36	40
50 to 67	47	46	47	47
Distribution of long-term SA days by sex	Col %	Col %	Col %	Col %
Men	43	34	44	46
Women	57	66	56	54
Ten most common diagnoses (total number of recipients over the study period)				
1	Major depressive disorder, single episode F32 (248,939)	Mononeuropathies of upper limb G56 (35,483)	Major depressive disorder, single episode F32 (248,939)	Reaction to severe stress, and adjustment disorders F43 (60,725)
2	Major depressive disorder, recurrent F33 (159,444)	Sleep disorders not due to a substance or known physiological condition F51 (24,830)	Major depressive disorder, recurrent F33 (159,444)	Other soft tissue disorders, not elsewhere classified M79 (20,468)
3	Other anxiety disorders F41 (158,884)	Acquired deformities of fingers and toes M20 (12,686)	Other anxiety disorders F41 (158,884)	Other joint disorder, not elsewhere classified M25 (16,466)
4	Dorsalgia M54 (88,247)	Acute upper resp infections of multiple and unspecified sites J06 (8246)	Dorsalgia M54 (88,247)	Fracture of foot and toe, except ankle S92 (12,476)
5	Osteoarthritis of knee M17 (78,129)	Excessive vomiting in pregnancy O21 (5169)	Osteoarthritis of knee M17 (78,129)	Internal derangement of knee M23 (11,589)
6	Reaction to severe stress, and adjustment disorders F43 (60,725)	Inguinal hernia K40 (4901)	Shoulder lesions M75 (58,052)	Fracture at wrist and hand level S62 (11,521)
7	Shoulder lesions M75 (58,052)	Overweight and obesity E66 (3941)	Thoracic, thoracolum, and lumbosacral intervertebral disc disorders M51 (52,105)	Injury of muscle, fascia and tendon at lower leg level S86 (7395)
8	Thoracic, thoracolum, and lumbosacral intvrt disc disorders M51 (52,105)	Female genital prolapse N81 (2627)	Malignant neoplasm of breast C50 (42,371)	Dislocation and sprain of joints and ligaments at ankle, foot and toe level S93 (7361)
9	Malignant neoplasm of breast C50 (42,371)	Cholelithiasis K80 (2371)	Bipolar disorder F31 (41,407)	Fibroblastic disorders M72 (6466)
10	Bipolar disorder F31 (41,407)	Hypertrophy of breast N62 (2203)	Osteoarthritis of hip M16 (38,905)	Sequelae of cerebrovascular disease I69 (6406)

*Note*: Col %: Column percentages.

The spring high cluster had slightly elevated SA recipient numbers in the spring and in December. This cluster consisted of 262 diagnostic groups and 1.8 million long-term SA recipients (81% of the analyzed long-term SA recipients) during the study period. The most common diagnostic groups were mental disorders and musculoskeletal disorders. Individuals under age 30 were more likely to be in this cluster than in the other two.

The autumn high cluster included 107 diagnostic groups and had higher numbers of long-term SA recipients from July to December. This cluster included stress-, injury-, and musculoskeletal disorder-related diagnostic groups, and consisted of 288,447 long-term SA recipients during the study period (13%).

## Discussion

This study showed that long-term SA followed a general seasonal pattern, with the number of recipients being lowest in the summer, and reaching three peaks in February–March, October, and December. This general pattern was observed for both men and women and for all age groups. Similar patterns in terms of less morbidity and mortality during summer than winter have been observed previously [25] but previous studies have lacked detailed nationwide data on SA.

This general pattern hid some meaningful variation across the diagnostic main chapters and more-specific diagnostic groups. For example, the observed seasonality was substantial for infections and respiratory system diseases and weaker for neoplasms. There was less seasonality in SA due to the diseases of the circulatory system, a finding which contrasts the studies on mortality and morbidity showing excess winter deaths and hospitalizations due to these causes [3,26]. Musculoskeletal and mental disorders, the two most important ICD-10 chapters in terms of SA in Finland, followed similar seasonal patterns with the highest number of recipients in December and lowest in August. While there are no comparable studies available, the findings regarding mental disorders are in line with an earlier study [18]. In terms of the impact of the COVID-19 pandemic, the ICD-10 chapter-specific trends were similar across the years, with the exception of spring 2020 when SA due to diseases in the respiratory system increased substantially.

There was heterogeneity in seasonality within the ICD-10 chapters. We therefore used cluster analysis to identify patterns of seasonality by 411 more-specific diagnostic groups. We used a data-driven approach to identify three seasonal patterns, which differed in terms of the sex and age of the recipients. The first cluster, winter and autumn peaks, consisted of a mix of diagnostic groups, and was more common in women. Non-organic sleep disorders was an important diagnostic group in this cluster. This seasonal pattern was similar to that reported in the study by Virtanen on common mental disorders, which was conducted before the COVID-19 pandemic [18]. The second cluster, spring high, consisted mainly of mental and musculoskeletal disorders. In the third cluster, recipient numbers were higher in the second half of the year.

This study did not provide evidence on the potential causal chains producing the observed patterns but it can be speculated that the causal factors are likely to be different for the different health conditions and clusters we identified. The first explanation is the differences in weather by season, including coldness and slipperiness during winter. The potential biological mechanisms of these weather aspects are reviewed in detail elsewhere [2,4,5]. Weather conditions also affect physical working environments, including, for example, working indoor and outdoor temperatures and slipperiness in the work place [27]. We did not merge our seasonal trend data with weather data to assess the role of weather. This is because we lacked more detailed geographical data. Subsequent investigations are needed to explore the role of variables reflecting different aspects of the weather.

Another often speculated explanation is differing daylight hours, which have been suggested to be particularly relevant for mental disorders [10]. Moreover, health care availability varies across the year. For some more severe health conditions, surgical and other complex non-urgent medical procedures may be conducted outside of holiday seasons. It is also possible that the patterns may be caused by changes in working conditions across the year. In many jobs, the end of the year, which showed the peak in SA, may be a stressful season. Finally, the observed patterns may reflect data artifacts. We did not have data on the denominator, that is, the total population at risk of SA, and we were therefore unable to calculate prevalence of SA. The implication of this is that the observed seasonality may reflect seasonal changes in the number of people in the workforce at risk of SA, for example, the sudden drop in January may be an indication of this.

The public health implication of these findings is that there is substantial potential to reduce SA at the population level by addressing the excess seasonality. Based on our calculations, if the number of SA recipients of each diagnostic group in every month of the year matched the lowest monthly level of this diagnostic group, SA would be reduced by some 19% in the 2023 population. For some diseases, seasonality may be unrealistic to reduce to this extent but for others there are readily available measures. These include, for example, supporting physical activity in late spring and winter in the form of subsidies to reduce mental disorder-related absence during this period.

A strength of this study is our use of an exceptionally large, nationwide set of comprehensive register data. We focused on long-term SA recipients only. Most SA spells are short, self-certified, and due to infections, which are generally covered by employers. We focused on long-term SA, which is covered by the social insurance system, and is often due to chronic diseases and sequelae. Shorter SA spells are likely to follow a different pattern. We did not have occupational class, area, or sector-aggregated data on long-term SA, even though they are significant determinants of long-term SA, and may indicate interesting patterns [28]. The ways in which seasons link to work ability health may be affected by working conditions [29,30]. Additional studies are needed to replicate these findings in other settings and to investigate how occupational status and sector and area differences influence the seasonality of long-term SA. The study was set in the Finnish context, in which both daylight and weather conditions vary considerably across the year; this could potentially induce some of the observed substantial variations in SA. Therefore, our findings may not be readily generalizable to other contexts. We did not exclude holiday periods in this study because people are generally eligible for SA during these periods in Finland. We also did not adjust for the compositional changes of the workforce. For example, some trends observed in the youngest age group may be due to their high share of seasonal work during summer periods. Due to data availability, we limited our analyses to those receiving SA on the 15^th^ of each month. This may overlook within-month trends and cause some noise due to measurement period.

In conclusion, these findings show that across years there are predictable seasonal patterns of long-term SA. These general trends are fairly similar across ages and sex but differ substantially by diagnostic group. However, the diagnostic group differences can be summarized to three ideal types. These findings suggest that occupational health practices should pay attention to injury prevention, particularly in the autumn, and should support people with sleeping disorders in the winter.

## Supplemental Material

sj-docx-1-sjp-10.1177_14034948251327545 – Supplemental material for Seasonal patterns of long sickness absence due to 411 diagnostic groups: a nationwide register-based study in Finland during 2020–2023 Supplemental material, sj-docx-1-sjp-10.1177_14034948251327545 for Seasonal patterns of long sickness absence due to 411 diagnostic groups: a nationwide register-based study in Finland during 2020–2023 by Aapo Hiilamo and Tea Lallukka in Scandinavian Journal of Public Health

## References

[1] Marinetti I Jdanov DA Jasilionis D , et al. Seasonality in mortality and its impact on life expectancy levels and trends across Europe. J Epidemiol Community Health, https://jech.bmj.com/content/early/2024/12/31/jech-2024-223050 (2024, accessed 10 January 2025). 10.1136/jech-2024-223050 PMC12171394 39740985

[bibr2-14034948251327545] Rau R. Seasonality in human mortality: a demographic approach. Heidelberg: Springer Science & Business Media, 2007.

[3] Suulamo U Remes H Tarkiainen L , et al. Excess winter mortality in Finland, 1971–2019: a register-based study on long-term trends and effect modification by sociodemographic characteristics and pre-existing health conditions. BMJ Open 2024;14:e079471. 10.1136/bmjopen-2023-079471 PMC10840061 38309756

[4] Gasparrini A Guo Y Hashizume M , et al. Mortality risk attributable to high and low ambient temperature: a multicountry observational study. Lancet 2015;386:369–75. 10.1016/S0140-6736(14)62114-0 PMC4521077 26003380

[5] Keatinge WR Coleshaw SR Cotter F , et al. Increases in platelet and red cell counts, blood viscosity, and arterial pressure during mild surface cooling: factors in mortality from coronary and cerebral thrombosis in winter. Br Med J (Clin Res Ed) 1984;289:1405–8. 10.1136/bmj.289.6456.1405 PMC1443679 6437575

[6] Healy JD. Excess winter mortality in Europe: a cross country analysis identifying key risk factors. J Epidemiol Community Health 2003;57:784–9. 10.1136/jech.57.10.784 PMC1732295 14573581

[7] Ledberg A. A large decrease in the magnitude of seasonal fluctuations in mortality among elderly explains part of the increase in longevity in Sweden during 20th century. BMC Public Health 2020;20:1674. 33167913 10.1186/s12889-020-09749-4 PMC7654045

[8] Törmälehto S Svirskis T Partonen T , et al. Seasonal effects on hospitalizations due to mood and psychotic disorders: a nationwide 31-year register study. Clin Epidemiol 2022;14:1177–91. 10.2147/CLEP.S372341 PMC9595069 36304786

[9] Lewy AJ Rough JN Songer JB , et al. The phase shift hypothesis for the circadian component of winter depression. Dialogues Clin Neurosci 2007;9:291–300. 17969866 10.31887/DCNS.2007.9.3/alewy PMC3202495

[10] Øverland S Woicik W Sikora L , et al. Seasonality and symptoms of depression: a systematic review of the literature. Epidemiol Psychiatr Sci 2019;29:e31. 10.1017/S2045796019000209 PMC8061295 31006406

[11] Knudsen AK Skogen JC. Monthly variations in self-report of time-specified and typical alcohol use: the Nord-Trøndelag Health Study (HUNT3). BMC Public Health 2015;15:172. 25884177 10.1186/s12889-015-1533-8 PMC4360933

[12] McCormack GR Friedenreich C Shiell A , et al. Sex- and age-specific seasonal variations in physical activity among adults. J Epidemiol Community Health 2010;64:1010–16. 10.1136/jech.2009.092841 19843499

[13] Titova OE Lindberg E Elmståhl S , et al. Seasonal variations in sleep duration and sleep complaints: a Swedish cohort study in middle-aged and older individuals. J Sleep Res 2022;31:e13453. 10.1111/jsr.13453 34355440

[14] van der Toorn JE Cepeda M Kiefte-de Jong JC , et al. Seasonal variation of diet quality in a large middle-aged and elderly Dutch population-based cohort. Eur J Nutr 2020;59:493–504. 30734846 10.1007/s00394-019-01918-5 PMC7058580

[15] Flinkkilä T Sirniö K Hippi M , et al. Epidemiology and seasonal variation of distal radius fractures in Oulu, Finland. Osteoporos Int 2011;22:2307–12. 10.1007/s00198-010-1463-3 20972668

[16] Salonen L Blomgren J Laaksonen M , et al. Sickness absence as a predictor of disability retirement in different occupational classes: a register-based study of a working-age cohort in Finland in 2007–2014. BMJ Open 2018;8:e020491. 10.1136/bmjopen-2017-020491 PMC5942421 29743328

[17] Kivimäki M Head J Ferrie JE , et al. Sickness absence as a global measure of health: evidence from mortality in the Whitehall II prospective cohort study. BMJ 2003;327:364. 12919985 10.1136/bmj.327.7411.364 PMC175810

[18] Virtanen M Törmälehto S Partonen T , et al. Seasonal patterns of sickness absence due to diagnosed mental disorders: a nationwide 12-year register linkage study. Epidemiol Psychiatr Sci 2023;32:e64. 10.1017/S2045796023000768 PMC7615330 37941381

[19] Botey Gaude L Cabrita J Eiffe FF , et al. Working conditions in the time of COVID-19: implications for the future, https://policycommons.net/artifacts/3184275/working-conditions-in-the-time-of-covid-19/3982888/ (2022, accessed 16 January 2025).

[20] Ervasti J Aalto V Pentti J , et al. Association of changes in work due to COVID-19 pandemic with psychosocial work environment and employee health: a cohort study of 24,299 Finnish public sector employees. Occup Environ Med 2022;79:233–41. 10.1136/oemed-2021-107745 34521683

[21] Schanzer DL Zheng H Gilmore J. Statistical estimates of absenteeism attributable to seasonal and pandemic influenza from the Canadian Labour Force Survey. BMC Infect Dis 2011;11:90. 21486453 10.1186/1471-2334-11-90 PMC3103439

[22] KELA. Sickness allowance, https://www.kela.fi/sickness-allowance (n.d., accessed 29 July 2024).

[23] Lorr M. Cluster analysis for social scientists, https://cir.nii.ac.jp/crid/1130282271216702208 (1983, accessed 20 May 2024).

[24] Rousseeuw PJ. Silhouettes: A graphical aid to the interpretation and validation of cluster analysis. J Comput Appl Math 1987;20:53–65.

[25] Akyeampong EB . Trends and seasonality in absenteeism. Perspectives on Labour and Income, https://www150.statcan.gc.ca/n1/pub/75-001-x/10607/9974-eng.pdf (2007, accessed 1 August 2024).

[26] Islam N Shabnam S Khan N , et al. Combinations of multiple long term conditions and risk of hospital admission or death during winter 2021–22 in England: population based cohort study. BMJ Med, https://bmjmedicine.bmj.com/content/3/1/e001016 (2024, accessed 17 January 2025). 10.1136/bmjmed-2024-001016 PMC11580288 39574426

[27] Karthick S Kermanshachi S Pamidimukkala A , et al. A review of construction workforce health challenges and strategies in extreme weather conditions. Int J Occup Saf Ergon 2023;29:773–84. 10.1080/10803548.2022.2082138 35622383

[28] Blomgren J Jäppinen S. Incidence and length of sickness absence among hierarchical occupational classes and non-wage-earners: a register study of 1.6 million Finns. Int J Environ Res Public Health 2021;18:501. 33435424 10.3390/ijerph18020501 PMC7827837

[29] Pajunen P Lönnqvist J Partonen T. Seasonal changes in mood and behavior in relation to work conditions among the general population. Scand J Work Environ Health 2007;33:198–203. 17572829 10.5271/sjweh.1128

[30] Hahn IH Grynderup MB Dalsgaard SB , et al. Does outdoor work during the winter season protect against depression and mood difficulties. Scand J Work Environ Health 2011;37:446–9. 10.5271/sjweh.3155 21359494

