# Supplementary material for: Seasonal patterns of long sickness absence due to 411 diagnostic groups: a nationwide register-based study in Finland during 2020–2023
Source: Scand J Public Health. 2025 Aug 11;54(6):624–31. doi: 10.1177/14034948251327545 (PMC13356261; doi:10.1177/14034948251327545)

Supplementary materials

Supplementary Figure 1. Optimal number of clusters by silhouette width. K-means clustering. Social Insurance Institution of Finland register data on all paid long-term SA recipients.
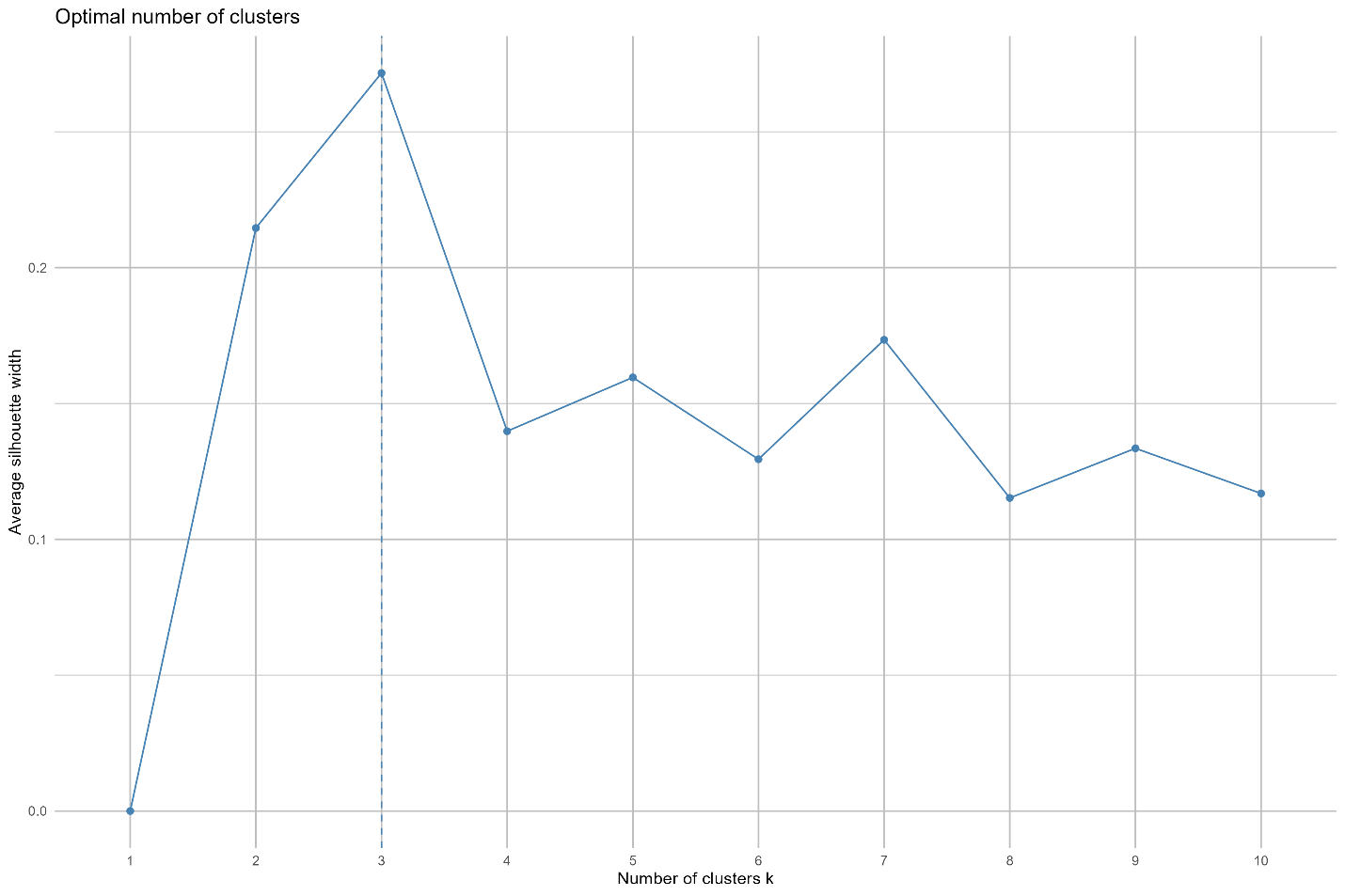


Supplementary Figure 2. Observed/expected long-term sickness absence (SA) recipients by year, month, and age in Finland in 2020–2023. Note that Y-axis is truncated. Social Insurance Institution of Finland register data.


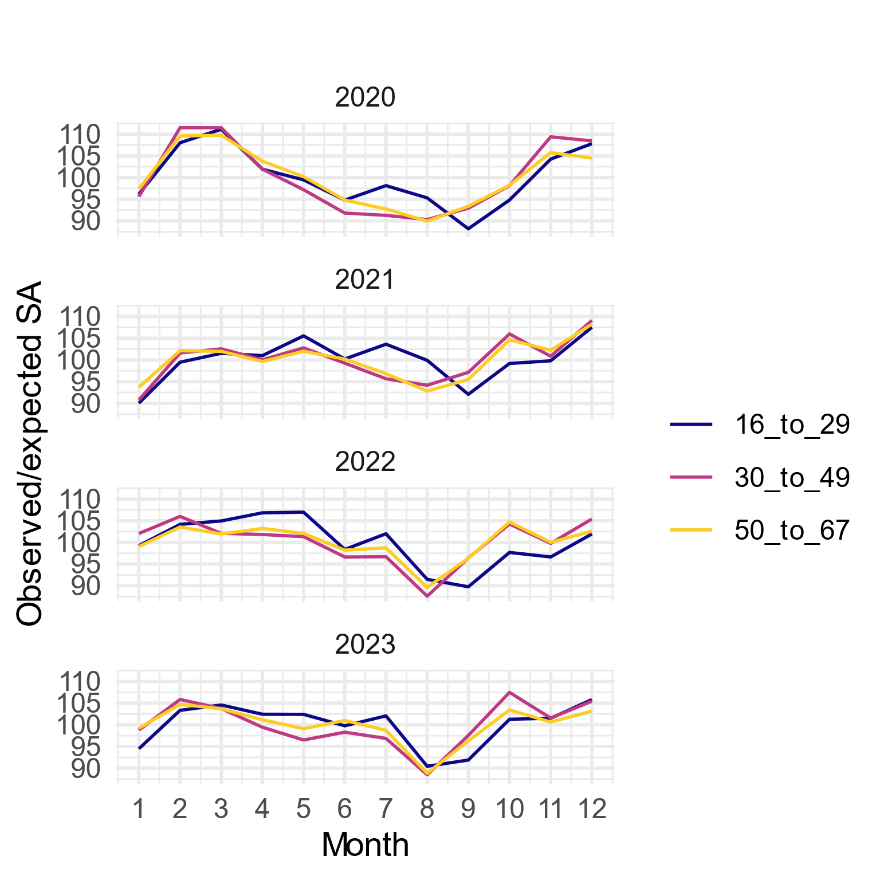


Supplementary Figure 3. Observed/expected long-term sickness absence (SA) recipients by year, month, and sex in Finland in 2020–2023. Note that Y-axis is truncated. Social Insurance Institution of Finland register data.


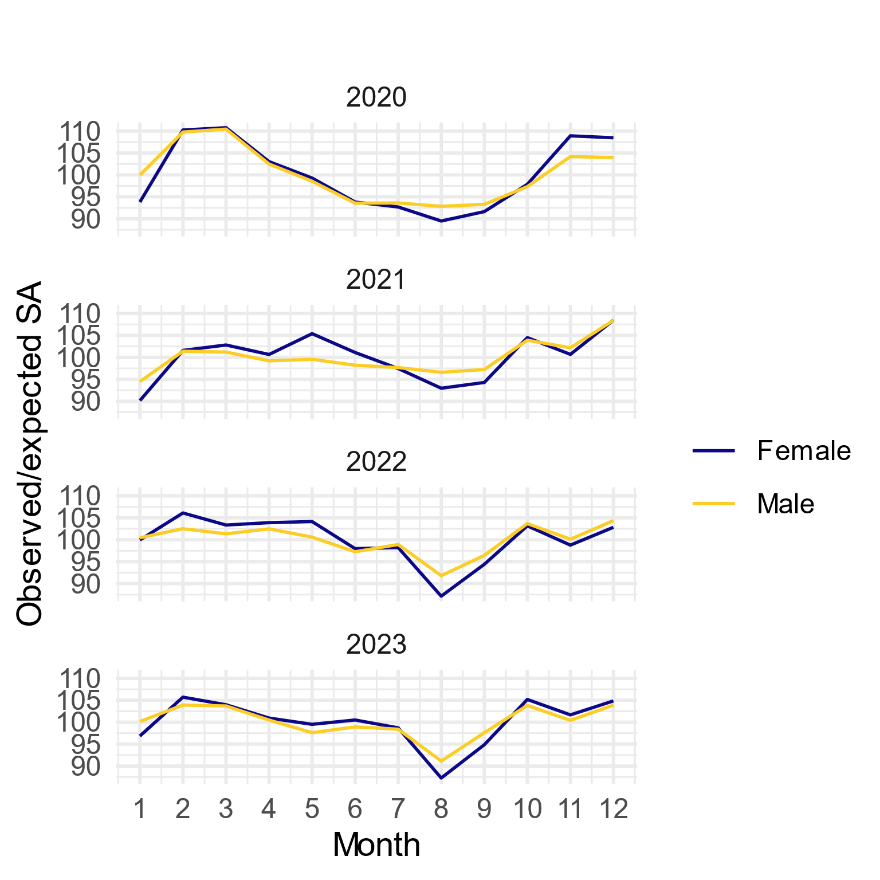

Supplement: sj-docx-1-sjp-10.1177_14034948251327545 – Supplemental material for Seasonal patterns of long sickness absence due to 411 diagnostic groups: a nationwide register-based study in Finland during 2020–2023 [file sj-docx-1-sjp-10.1177_14034948251327545.docx]
